# Management of pediatric pleural empyema: a national survey of pediatric surgeons in Brazil

**DOI:** 10.36416/1806-3756/e20230318

**Published:** 2024-05-08

**Authors:** Felippe Flausino, Luiza Maes Manara, Bruna Baioni Sandre, Gilson Nagel Sawaya, Rosemeri Maurici

**Affiliations:** 1. Departamento de Cirurgia Pediátrica, Hospital Infantil Joana de Gusmão, Florianópolis (SC) Brasil.; 2. Departamento de Radiologia Pediátrica, Hospital Infantil Joana de Gusmão, Florianópolis (SC) Brasil.; 3. Departamento de Cirurgia Pediátrica, Faculdade de Medicina, Pontifícia Universidade Católica de Campinas, Campinas (SP) Brasil.; 4. Departamento de Clínica Médica, Universidade Federal de Santa Catarina - UFSC - Florianópolis (SC) Brasil.; 5. Programa de Pós-Graduação em Ciências Médicas, Universidade Federal de Santa Catarina - UFSC - Florianópolis (SC) Brasil.

**Keywords:** Empyema, pleural, Thoracic surgery, video-assisted, Fibrinolysis, Surveys and questionnaires, Pediatric surgery

## Abstract

**Objective:**

To identify how pediatric surgeons manage children with pneumonia and parapneumonic pleural effusion in Brazil.

**Methods:**

An online cross-sectional survey with 27 questions was applied to pediatric surgeons in Brazil through the Brazilian Association of Pediatric Surgery. The questionnaire had questions about type of treatment, exams, hospital structure, and epidemiological data.

**Results:**

A total of 131 respondents completed the questionnaire. The mean age of respondents was 44 ± 11 years, and more than half (51%) had been practicing pediatric surgery for more than 10 years. The majority of respondents (33.6%) reported performing chest drainage and fibrinolysis when facing a case of fibrinopurulent parapneumonic pleural effusion. A preference for video-assisted thoracic surgery instead of chest drainage plus fibrinolysis was noted only in the Northeast region.

**Conclusions:**

Chest drainage plus fibrinolysis was the treatment adopted by most of the respondents in this Brazilian sample. There was a preference for large drains; in contrast, smaller drains were preferred by those who perform chest drainage plus fibrinolysis. Respondents would rather change treatment when facing treatment failure or in critically ill children.

## INTRODUCTION

Pleural effusion is a serious complication of pneumonia in children. Although pleural effusion is rare, it is responsible for almost half of hospital admissions in Brazil.^1^ ^,^ ^2^ Parapneumonic pleural effusion (PPE) has a three-stage evolution, and its treatment depends on the phase in which it is found. In the beginning, the increase in capillary permeability promotes an accumulation of free fluid in pleural space. The infectious process leads to fibrin and septal formation in the pleural space. In this phase, known as fibrinopurulent, it is possible to identify these loculations or septations on chest ultrasound.^3^ This is a good method to confirm the presence of pleural fluid and to characterize the nature of a PPE and guide management. It was shown that classifying the stage of PPE and individualizing the treatment leads to shorter length of hospital stay.^4^ The treatment of PPE is carried out according to the quality of the pleural fluid.^5^ ^,^ ^6^ The gold standard of treatment is not defined and may depend on medical and equipment availability, as well as the experience of some centers. Chest drainage and administration of a fibrinolytic agent promote evacuation of pleural cavity and pulmonary expansion.^5^ ^,^ ^6^ In several countries, the association of chest drainage and administration of fibrinolytic agents is the first line treatment. In Brazil, the number of centers using chest drainage and fibrinolysis in these patients is unknown. However, video-assisted thoracoscopic surgery (VATS) is routinely preferred, because it allows adequate visualization of the effusion and helps position the chest drain. Many surgeons have conducted it according to the availability of thoracoscopic equipment.^7^ ^,^ ^8^

Necrotizing pneumonia may be difficult to diagnose on chest radiography in its initial phase, although it may show cavitations (pneumatoceles) later. Chest CT is not routinely recommended for evaluating empyema, but it could be used to detect parenchymal lung abnormalities, endobronchial obstruction, lung abscesses, and mediastinal abnormalities.^9^

Therefore, it is necessary to know and establish protocols for the treatment of empyema in children. Surveys can help answer several questions related to pediatric surgeons’ practice in managing pleural empyema.^10^ An international survey applied to physicians who treat pleural empyema in adults identified that the practices adopted were very divergent. There was also a wide variation in fibrinolytic dosing.^11^ This study aimed to identify how PPE in children is managed by means of an online questionnaire applied to pediatric surgeons in Brazil.

## METHODS

The Research Ethics Committee of *Hospital Infantil Joana de Gusmão* approved the study (CAAE no. 58259922.8.0000.5361). A pilot survey with 29 questions was developed and randomly applied to 10 regional pediatric surgeons in the city of Florianópolis, Brazil. After the pilot, the researchers have selected a total of 27 questions, and the definitive questionnaire was applied to pediatric surgeons participating in the Brazilian Association of Pediatric Surgery mailing system between February and March of 2023. Google Forms was chosen to produce and distribute the questionnaire. All of the respondents were an active member of the Association. Invitations to participate in the survey were sent via e-mail sent by the Association. Participants who did not complete the questionnaire within 21 days were contacted again. Descriptive analysis was performed using absolute data and reported as absolute and relative frequencies. Continuous variables were reported as medians or means. The Shapiro-Wilk test was used for the evaluation of variables with normal distribution. Categorical variables were evaluated using the chi-square test, and numerical variables were evaluated using the t-test. The Cramer’s V test was used to assess the effect size. ANOVA was performed to compare means and categorical variables. The chi-square test and the Fisher’s exact test were used for comparative analysis and the value of p < 0.05 was considered significant for testing the hypotheses.

## RESULTS

There were 131 respondents who completed the questionnaire. Table 1 shows the distribution of responses. The Shapiro-Wilk test demonstrated normal distribution of the responses. The mean age of respondents was 44 ± 11 years, and 67 (51%) of the respondents had been practicing pediatric surgery for more than 10 years. When asked about the frequency of being called to evaluate children with pleural effusion, the majority answered that this always or almost always happened (61.8%). More than half of respondents stated that they had treated 16 or more children within the last 12 months.

**Table 1:** Characteristics of the respondents (N = 131).

Characteristic		
Age, years (mean ± SD)	44 ± 11	
	n	%
Gender		
Male	59	45,0
Female	72	55,0
Region		
North	5	3.8
Northeast	31	23.7
Central-West	17	13,0
South	29	22.1
Southeast	48	36.6
Effusion stage at admission		
I - Exudative	37	28.2
II - Fibrin-purulent	90	68.7
III - Organized	3	2.3
Unknown	1	0.8
Treatment		
Chest drainage with or without saline	41	31.3
Chest drainage plus fibrinolysis	44	33.6
VATS	35	26.7
Thoracotomy	3	2.3
Other	8	6.1
Chest drain size		
Small drain (≤ 14Fr)	22	16.8
Large drain (≥ 16Fr)	99	75.6
Pigtail	4	3.1
Other	6	4.6
Time of practice		
Less than 5 years	37	28.2
Between 6-10 years	30	19.8
Between 11-20 years	4	21.4
More than 20 years	30	30.5

VATS: video-assisted thoracic surgery.

**Figure 1:**
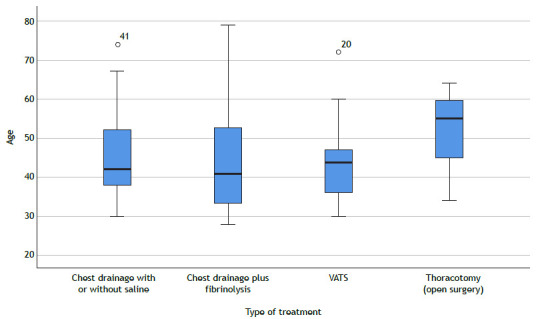
Type of treatment selected by age of respondents (mean age: black line in the rectangle). VATS: video-assisted thoracic surgery.

The correlation of chest drain size, Brazilian macroregion, treatment in case of illness, and type of treatment is shown in Table 2. No significant differences were found regarding respondents’ mean age and type of surgical treatment of patients for fibrinopurulent PPE (ANOVA: 218.673; df: 4; p = 0.7). However, the mean age of respondents (51 years) who performed thoracotomy was higher than that of those who performed other types of treatment, as shown in Figure 1.

**Table 2:** Correlations between chest drain size, macroregions of Brazil, and types of treatment.

Treatment							
Variable	Thoracostomy plus saline solution	Thoracostomy plus fibrinolytic agent	VATS	Thoracotomy	Total	Cramer’s V	p*
Drain size							
Small drain (≤ 14Fr)	3 (14.3%)	15 (71.4%)	3 (14.3%)	0 (0,0%)	21 (16.2%)	Moderate (0.251)	0.07
Large drain (≥ 16Fr)	34 (36.5%)	25 (26.8%)	31 (33.3%)	3 (3.2%)	93 (70.9%)		
Pigtail	0 (0.0%)	3 (75.0%)	1 (25.0%)	0 (0.0%)	4 (3.1%)		
Other	4 (80.0%)	1 (20.0%)	0 (0.0%)	0 (0.0%)	5 (3.8%)		
Valid responses					131 (100%)		
Change in treatment							
Yes, if VATS	11 (61.1%)	4 (22.2%)	0 (0.0%)	3 (16.7%)	18 (14.6%)	Moderate (0.459)	< 0.01
Yes, if fibrinolytic agent	22 (48.9%)	1 (2.2%)	22 (48.9%)	0 (0.0%)	45 (36.6%)		
No	7 (11.9%)	39 (66.1%)	13 (22.0%)	0 (0.0%)	59 (48.0%)		
Valid responses					122 (100%)^a^		
Different treatment if unstable							
Yes, always	12 (44.4%)	5 (18.5%)	8 (29.6%)	2 (7.4%)	27 (24.8%)	Weak (0.231)	0.07
No, only in necrosis	12 (26.1%)	18 (39.1%)	15 (32.6%)	1 (2.2%)	46 (42.2%)		
No	14 (38.9%)	17 (47.2%)	5 (13.9%)	0 (0.0%)	36 (33.0%)		
Valid responses					109(100%)^a^		
Region							
North	1 (33.3%)	2 (66.7%)	0 (0.0%)	0 (0.0%)	3 (2.8%)	Moderate (0.282)	< 0.01
Northeast	15 (60.0%)	0 (0.0%)	8 (32.0%)	2 (8.0%)	25 (23.1%)		
Center-west	5 (35.7%)	7 (50.0%)	2 (14.3%)	0 (0.0%)	14 (13.0%)		
Southeast	14 (35.0%)	20 (50.0%)	6 (15.0%)	0 (0.0%)	40 (37.0%)		
South	3 (11.5%)	10 (38.5%)	12 (46.2%)	1 ( 3.8%)	26 (24.1%)		

VATS: video-assisted thoracic surgery. *Chi-square test and Fisher’s exact test. ^a^Some missing data.

Pediatric thoracic surgeries represented less than 25% of the surgeries performed by most respondents. Only 11 respondents used more than 50% of their working time with pediatric thoracic surgery. Only 21.4% of respondents reported not performing other thoracoscopic procedures. Stage II pleural effusion was treated only with chest drainage, with or without saline, by 68% of the participating pediatric surgeons.

### Intervention

Most of the patients were hospitalized with fibrinopurulent PPE according to 68.7% of the respondents. Additionally, the most common surgical treatment was chest drainage followed by the administration of fibrinolytics (32.8% of respondents), and the most commonly used fibrinolytic was alteplase (in 97%). Nevertheless, 31.3% of respondents reported that they performed chest drainage with or without the use of saline solution.

The surgeon or resident was responsible for administering the fibrinolytic agent in 88.3% of the responses. None of the respondents reported that interventional radiologists had administrated fibrinolytics.

When asked about the size of the thoracic drain, there was a preference for using larger drains (in 75%). However, those who reported to perform thoracostomy and fibrinolysis were associated with the use of small drains (< 14 Fr; Fisher’s: 22.947; df: −12; p < 0.001; Cramer’s V: 0,251). The analysis of the adjusted residuals indicated that, for the group who reported using chest drainage and fibrinolysis (CDF), there was a higher than expected frequency regarding the use of small drains.

We also asked the participants that if they had other treatment options available, they would adopt them. In the CDF group, 88.6% would not change their conduct if they had other treatment methods. On the other hand, those who would change if they had fibrinolytics available were mostly those who reported using VATS or drainage plus saline solution. (Fisher’s: 84.031; gL : 12; p < 0.001).

**Figure 2:**
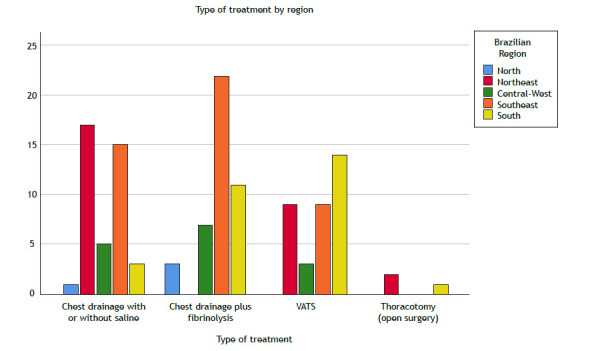
Number of respondents by Brazilian macroregion and type of treatment selected. VATS: video-assisted thoracic surgery.

When compared by Brazilian macroregions, many of the respondents in the Northeast performed chest drainage with or without saline (n = 17; 41%), as shown in Figure 2. Meanwhile, the Southeast region had the highest number of respondents who performed VATS (40%).

None of the respondents in the Northeast region performs CDF. There was an association between respondents who were in the Northeast and performed thoracostomy and pleural drainage with or without saline. The Southern region had the highest number of respondents who performed VATS.

### Complications and treatment failure

Therapeutic failure was considered as the persistence of fever, need for supplemental oxygen, and lack of appetite for 51.5% of respondents. Most respondents (66.4%) chose another surgical treatment when a therapeutic failure was considered.

When asked whether the patient should be treated differently if he/she was clinically ill, 28 (21.4%) of respondents reported that they agreed with the change, 49 (37.4%) replied that they would treat him/her differently if there was pulmonary necrosis, and another 38 (29.0%) said that they would not change treatment.

Almost half of the respondents reported having followed the children regardless of the treatment performed. When there is pulmonary necrosis, 61 (46.6%) of respondents stated that they would maintain the conduct according to the treatment of the pleural disease; however, another 29 (22.1%) answered that they would perform VATS, and 34 (26%) said they would perform thoracotomy to manage pulmonary necrosis. Surgery for bronchopleural fistula was said to be carried out mostly when the fistula is persistent (60% of respondents). In contrast, 41 (31.3%) of respondents would not choose a surgical procedure when facing a bronchopleural fistula.

## DISCUSSION

The result of this survey shows that CDF is the most common treatment adopted in Brazil (n = 44; 33.6%). However, 41 respondents (31.3%) reported performing chest drainage with or without saline solution. One randomized trial found a reduction in length of hospital stay comparing the use of urokinase and saline in children.^12^ In adults, another study reported a lower risk of treatment failure using fibrinolytics.^13^ Treatment for fibrinopurulent PPE using CDF as the first line treatment and VATS in case of failure are recommendations of the British Thoracic Society (BTS) and the American Pediatric Surgical Association.^5^ ^,^ ^6^ Various centers have adopted this type of approach. However, 35 (26.7%) of respondents preferred VATS as the first line treatment for fibrinopurulent PPE. In 2017, a systematic review^14^ stated that VATS can reduce the length of hospital stay when compared with thoracostomy and drainage alone by almost 3 days. The evaluation of the use of fibrinolytics in that review was disadvantaged by insufficient evidence. Thoracotomy was also superior to thoracostomy and pleural drainage in terms of length of hospital stay in children.^14^

St Peter et al.^15^ performed a randomized clinical trial with 36 participants, comparing fibrinolysis with alteplase and VATS, and found equal length of hospital stay and drainage time; however, the costs in the VATS group were more expensive. Sonnappa et al.,^16^ in a randomized clinical trial involving 60 participants, compared fibrinolysis using urokinase with VATS and found no differences in terms of mean length of hospital stay and number of days for chest drain removal. The abovementioned systematic review^14^ showed no difference in mortality between surgical or nonsurgical treatment.

Administration of alteplase has an advantage over VATS, because it is less invasive and does not require general anesthesia. Nevertheless, a retrospective study found advantages in applying a fibrinolytic (urokinase) after thoracoscopy performed with moderate sedation.^17^ However, the medication used (propofol+remifentanil+sevoflurane) might be considered as general anesthesia in some centers.

There is no evidence to determine which treatment for fibrinopurulent PPE in children is the best. The use of CDF, VATS, or chest drainage alone in Brazil might result from the unavailability of thoracoscopic equipment or fibrinolytic agents. Certainty, most of the participants who chose CDF (n = 39; 88.6%) stated that they would not change the treatment if VATS was available. In contrast, those who reported using VATS or chest drainage with or without a fibrinolytic-22 (62%) and 22 (53%), respectively-stated that they would change treatment if a fibrinolytic agent was available (Fisher: 84.031; gL: 12; p < 0.001; Cramer’s V: 0,463).

Large drains were preferred by most of the respondents (75%), but those who chose CDF used smaller drains. An open randomized study^18^ with 130 children compared drainage of empyema with small catheters (less than 14Fr) and large catheters (greater than 14Fr) and found no differences between length of hospital stay, chest drainage time, and volume drained. They also concluded that smaller drains caused less pain without any impact on the clinical outcome.^18^ The BTS guidelines recommend the use of small drains (including pigtail catheters) to minimize patient discomfort.^6^

Brazil is a continental country with inequitable distribution of investments in health. Besides that, income inequality could affect the management of some cases of PPE. There was an association with the macroregion of Brazil and the type of intervention. The Fisher’s exact test showed significance for those who reported being in the Northeast and treating for PPE with drainage and saline solution in comparison with those in other regions (p < 0.001). In addition, there was an association of those who responded being in the South and treating for PPE with VATS (p < 0.001; Cramer’s V: 0.282). On the other hand, there was an association between those who reported they would change treatment if VATS was available and being in the Northeast (p = 0.04; Cramer’s V: 0.216). Some authors from developing countries reported no significant differences between treatment with CDF and with VATS in their centers. In a randomized trial with 41 children,^19^ the use of fibrinolytic agents were not inferior to VATS in the Indian subcontinent. In addition, the need of blood transfusion was significantly higher in the VATS group in that study.^19^ To sum up, VATS might require more medical equipment (hospital apparatus), and it is difficult to compare surgical management between different countries or centers with different number of patients or different health care structures.

Failure in the treatment of empyema is considered when fever, prolonged supplemental oxygen requirement, and loss of appetite occur. The majority of respondents agreed with that statement. Most of them chose another type of treatment when facing failure. There is a preference for surgical treatment (VATS or thoracotomy) when there is treatment failure or necrosis. Indeed, consideration for VATS after chemical debridement should occur when the patient is persistently ill after chest tube drainage diminishes and imaging proves substantial disease in pleural space.^5^ Meanwhile, some authors showed that, instead of performing VATS, it would be possible to do an additional course of fibrinolysis or replace the chest drain in other non-contemplated loculations in order to avoid surgery in children with symptomatic pleural disease.^20^

Dealing with the experience in chest ultrasound, 71% of respondents reported not being experienced. However, 91.6% of respondents reported using chest CT for the diagnosis of pulmonary necrosis. Chest ultrasound is portable, easy to perform, and involves no radiation. Likewise, it is superior to chest CT to show pleural problems.^21^ The ultrasonographic aspect of a pleural effusion should indicate the treatment and can be carried out by the surgical team. The use of point-of-care chest ultrasound should be encouraged to improve management of pleural empyema. The BTS guidelines advise the use of ultrasound to guide thoracentesis and to identify the best place for drain insertion.^6^ In addition, ultrasound could be used before thoracoscopy to assess the best site of trocar insertion according to presence, quantity, and characteristics of pleural effusion.^22^

It is estimated that there are 1,720 pediatric surgeons registered in Brazil and probably less than half of this number is not active in the specialty, although we only obtained 131 responses.^23^ The low rate of respondents was a limitation. The interest in responding may represent a biased sample of those surgeons who perform most thoracic procedures and might not represent reality. Thoracic surgeons and pediatric pulmonologists are responsible for caring for children with pleural effusion, and they were not included in this survey, which might be a bias.

In a nutshell, chest drainage with fibrinolysis is the treatment adopted by most of the respondents in Brazil. Besides that, VATS could be more used if it is widely available. Moreover, there was a preference for using larger drains in this sample. The majority of respondents would prefer to do another procedure when facing treatment failure. The point-of-care chest ultrasound should routinely be used in the diagnosis and selection of treatment strategies.
